# Supplementary material for: Electronic correlations and transport in iron at Earth’s core conditions
Source: Nat Commun. 2020 Aug 14;11:4105. doi: 10.1038/s41467-020-18003-9 (PMC7429499; doi:10.1038/s41467-020-18003-9)
Supplement: Supplementary file 1 — Supplementary Information [file 41467_2020_18003_MOESM1_ESM.pdf]

Supplementary Information for
Electronic correlations and transport in iron at Earth's core conditions
by Pourovskii *et al.*

I. SUPPLEMENTARY NOTE 1: INFLUENCE OF THE SUPERCELL SIZE

In Supplementary Figure 1 we compare the thermal conductivity computed for our sets of 2×2×2 and 3×3×3 distorted bcc supercells (SCs) using the site and orbital averaged DMFT self-energy $\langle\Sigma\rangle$, see Fig. 2a and 2b of the main text, as well as Methods for calculational details. One may see that the magnitude of conductivity decreases a little bit (by about 6%) with the increasing SC size.

The conductivites for the perfect 2×2×2 and 3×3×3 bcc SCs calculated using the same average self-energy $\langle\Sigma\rangle$ are also shown in the same figure; they are higher than those obtained for the perfect 2×2×2 in real DFT+DMFT calculations (the red hashed square in Fig. 1b). The reason is, of course, that the correlations are reduced by lattice distortions as explained in the main text, see Figs. 2 and 3 of the main text and the discussion therein.

In Supplementary Figure 2 we show the electron-lattice-scattering (ELS) optical conductivity $\sigma_{\mathrm{el-lat}}$ as well as the ELS dynamical thermal conductivity $\kappa_{\mathrm{el-lat}}(\omega)$ calculated within DFT by the method of refs. 31 and 32 vs. the size of simulation SCs. The DC conductivity is obtained by taking the $\omega \to 0$ limit. One sees that the result for 3×3×3 SCs is very close to that for the largest considered SCs (5×5×5), while the one obtained with 2×2×2 SCs still deviates from it and exhibits significant noise.

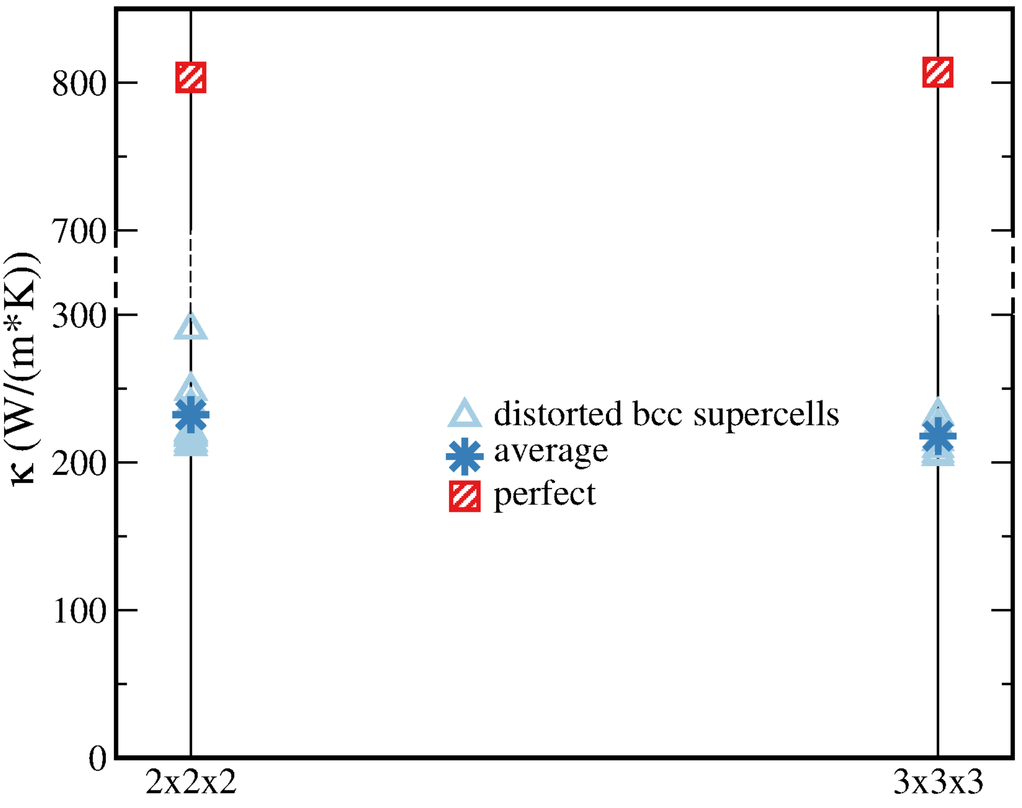

SUPPLEMENTARY FIGURE 1. Thermal conductivities for the sets of 2×2×2 and 3×3×3 bcc supercells obtained with the site and orbital averaged self-energy $\langle\Sigma\rangle$ and double-counting correction inserted into their converged DFT band-structures as described in the Methods section of the main text. The meaning of symbols is the same as in Fig. 1 of the main text

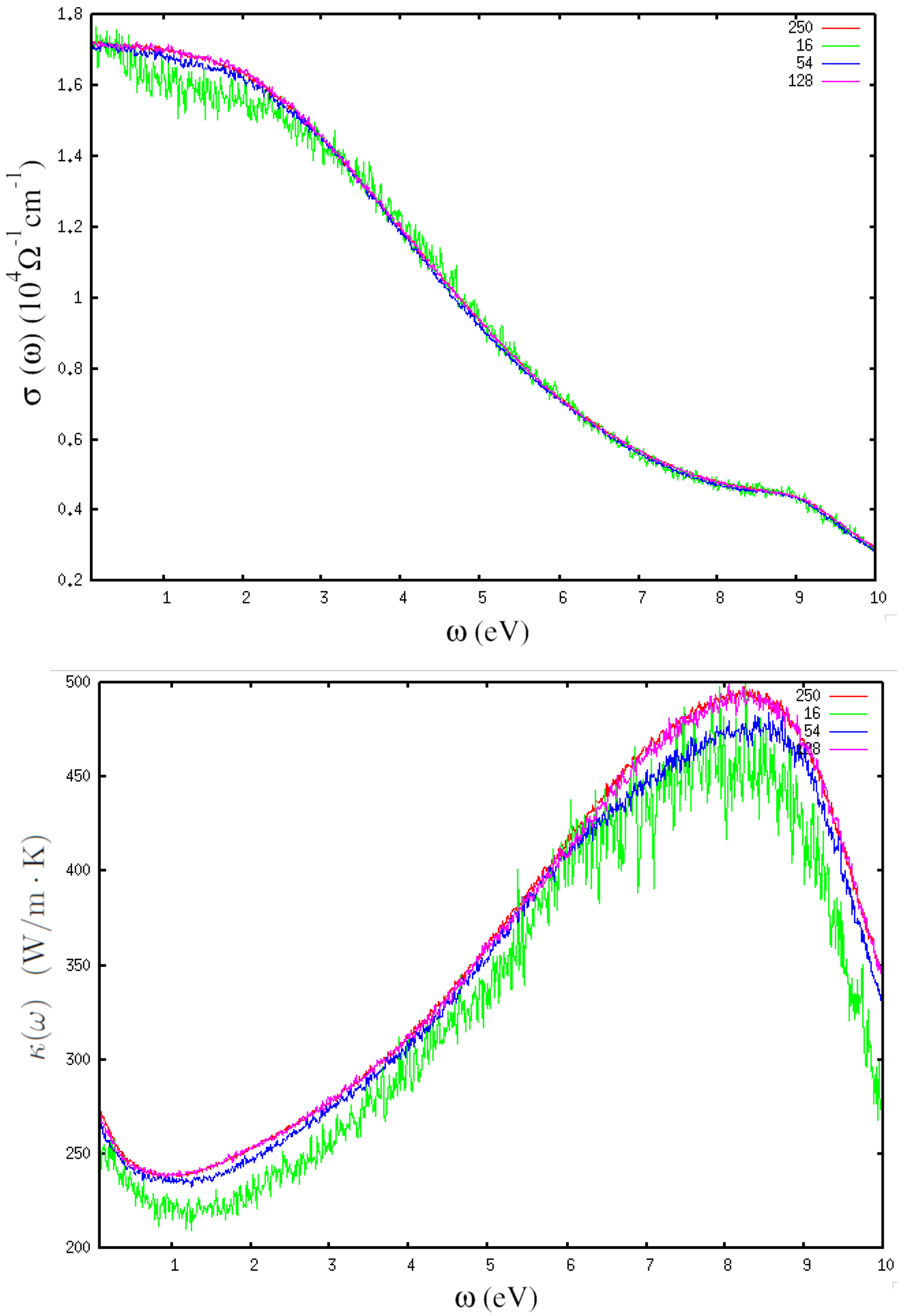

SUPPLEMENTARY FIGURE 2. Electron-lattice-scattering (ELS) optical conductivity (upper panel) and dynamical thermal conductivity (lower panel) calculated within DFT using bcc supercells (SC) of different sizes. The DC conductivity is obtained by taking the $\omega \to 0$ limit. One sees that the values obtained using 3×3×3 54-atoms SCs are very close to final results with 5×5×5 250-atoms SCs.
